# From guidelines to practice: an Egyptian expert opinion on type 2 diabetes mellitus management in primary care settings

**DOI:** 10.1186/s12875-025-03091-7

**Published:** 2025-11-27

**Authors:** Mohamed Khattab, Samir H. Assaad-Khalil, Atef Bassyouni, Farid Fawzy, Hesham El Gayar, Ibrahim Elebrashy, Khaled Elsayed Elhadidy, Khalifa Abdallah, Mohamed Hesham El Hefnawy, Mohamed Reda Halawa, Nabil Elkafrawy, Samir George, Yehia Ghanem

**Affiliations:** 1 Department of Internal Medicine and Diabetes, Faculty of Medicine, Cairo University, Cairo, Egypt; 2 Department of Internal Medicine, Unit of Diabetes, Lipidology, and Metabolism, Faculty of Medicine, Alexandria University, Alexandria, Egypt; 3 National Institute of Diabetes and Endocrinology, Cairo, Egypt; 4 Department of Diabetes & Endocrinology, Faculty of Medicine, Zagazig University, Sharkeya, Egypt; 5 Department of Endocrinology, Faculty of Medicine, Ain Shams University, Cairo, Egypt; 6 Department of Internal Medicine, Diabetes, Endocrinology and Metabolism Unit, Faculty of Medicine, Beni Suef University, Beni Suef, Egypt; 7 Department of Internal Medicine, Unit of Diabetes and Endocrinology, Faculty of Medicine, Menoufia University, Menoufia, Egypt

**Keywords:** Diabetes mellitus, Prediabetes, Insulin, Glycemic, Expert opinion

## Abstract

The broad classification of diabetes includes type 1 diabetes, type 2 diabetes, gestational diabetes, and other specific types such as monogenic diabetes. Additionally, prediabetes represents an at-risk state that requires early recognition. The incidence of type 2 diabetes mellitus (T2DM) has risen over the years, necessitating continuous medical care from well-trained staff. Accurate classification of diabetes is crucial for determining the appropriate treatment approach for each case. While this expert panel opinion specifically addresses management of T2DM, awareness of other categories is important to avoid misclassification and ensure timely referral. The diagnosis of diabetes hinges on the measurement of blood glucose levels, with specific thresholds defining both prediabetes and diabetes. Early diagnosis and proper diabetes treatment can prevent or delay the onset of diabetes-associated complications, whereas misdiagnosis or delayed diagnosis makes treatment decisions more challenging. Additionally, co-morbidities often require adjustments to standard management approaches. Junior and less experienced healthcare providers (HCPs) may struggle with these complexities, highlighting the need for clear guidance and structured support in clinical decision-making. A panel of 13 Egyptian experts analyzed global guidelines and challenges in managing diabetes in Egypt. Through a structured discussion, they formulated a streamlined resource for HCPs, outlining treatment strategies, comorbidity considerations, and referral guidelines to enhance the quality of care for people with diabetes. This expert opinion covers the following topics: (a) treatment options and glycemic targets, (b) insulin regimens, (c) T2DM associated with cardiovascular disease, and (d) diabetic kidney disease. This expert opinion provides a streamlined guide for HCPs, particularly those in primary care, to enhance diabetes management in Egypt. Adoption of such guideline-based recommendations is crucial to standardize care, improve glycemic outcomes, and reduce the long-term burden of complications. Implementing these recommendations may help mitigate diagnostic delays, optimize patient care, and reduce diabetes-related complications.

**Supplementary Information:**

The online version contains supplementary material available at 10.1186/s12875-025-03091-7.

## Introduction

Diabetes Mellitus (DM) is a common endocrine disorder and one of the top 10 causes of death worldwide [1]. Recently, there has been a marked increase in the incidence and prevalence of DM that has almost reached epidemic proportions. Diabetes is broadly classified into type 1 diabetes, type 2 diabetes, gestational diabetes, and other specific types (including monogenic diabetes, such as MODY and neonatal diabetes). Prediabetes, characterized by impaired fasting glucose or impaired glucose tolerance, is an important intermediate state that often precedes type 2 diabetes mellitus (T2DM). Among these categories, T2DM is a major public health challenge and by far the most prevalent in Egypt, with its incidence rising markedly over the last decades [2]. According to the International Diabetes Federation (IDF) Diabetes Atlas 2025, an estimated 19.8% of Egyptian adults aged 20–79 years are living with diabetes, with an age-adjusted comparative prevalence of 22.4%. Importantly, more than 62% of cases remain undiagnosed, suggesting that the true burden is substantially higher than reported [3].

T2DM is the predominant form of diabetes, constituting the majority of cases [4]. Its multifactorial pathophysiology encompasses a complex interplay between insulin resistance, beta-cell dysfunction, incretin deficiency, dysregulation of hepatic glucose production, adipose tissue dysfunction, chronic inflammation, and oxidative stress. Understanding these underlying mechanisms is the basis for developing targeted therapeutic interventions and enhancing patient care [5].

Given the significant effects of DM on various body systems, causing serious health issues, it is crucial to establish a systematic healthcare approach for people living with diabetes. Best practice for diabetic patients requires a multidisciplinary team that includes primary care physicians, endocrinologists, nephrologists, and cardiologists [6]. Equally important are non-physician healthcare professionals such as dietitians, diabetes care and education specialists, podiatrists, and exercise physiologists, who provide essential lifestyle and self-management support [7]. Podiatrists and psychologists may also play key roles, alongside other specialists such as neurologists, vascular surgeons, and ophthalmologists, in addressing complications and psychosocial aspects of care [8–10].

Glycemic control among Egyptian people with diabetes is suboptimal [11]. The International Diabetes Management Practices Study (IDMPS, Wave 8) reported that 82.6% of Egyptian patients had HbA1c levels ≥ 7%, with rural clinics reporting up to 92.3% of patients not meeting glycemic targets. Broader reviews of Egyptian studies show that the percentage of patients above HbA1c targets ranges from 45.2% to 93% [12]. This could lead to potential multisystem complications, which negatively impact the patient’s quality of life, increase morbidity and mortality, and raise costs. A recent study conducted in Canada showed that early referral of diabetic patients to specialized physicians improves the likelihood of achieving glucose targets and reduces cardiovascular risk [13]. In Egypt, most people with diabetes are managed in primary care settings. Notably, comparable challenges are observed in the broader North African region: for example, in Tunisia, only 15.5% of patients with type 1 diabetes and 24.7% of those with type 2 diabetes achieved the recommended HbA1c goal of < 7% [14]. Furthermore, in severely resource-limited areas of North Africa, glycemic control has been reported as uniformly above target [15]. A similar pattern is seen in other African settings, where only about one-third of patients meet glycemic targets [16]. In the absence of national guidelines for T2DM management in Egypt, strengthening primary care physicians’ knowledge and practice, along with ensuring timely referral, is crucial to improving patient outcomes and reducing the burden of diabetes-related complications. Indeed, recent data underscore the severity of this issue: in private-sector individuals with diabetes, only 18.4% achieved the HbA1c target of ≤ 7% [17]. In rural primary care clinics, not meeting glycemic targets was reported in 92.3% of subjects [18]. A broader review of Egyptian T2DM studies showed that HbA1c levels above target ranged from 45.2% to 93% [11]. These findings underscore the urgent need to generate locally relevant recommendations to support Egyptian primary care physicians in managing T2DM. International guidelines are comprehensive but often difficult to translate into practice in resource-constrained settings. Their inconsistent adoption into routine practice remains a major barrier. The importance of adopting guideline-based management lies in its ability to harmonize care across providers, reduce clinical inertia, and ensure that patients receive therapies proven to prevent complications. For Egypt, where T2DM prevalence and complication rates are high, guideline adoption represents a critical step toward improving population-level outcomes.

In this article, we aim to contextualize the global recommendations, tailor them to the Egyptian healthcare environment, and provide a simplified expert panel opinion-based summary of diabetes management, with specific adaptation to the Egyptian context. Our recommendations draw upon international guidelines as well as Egyptian data and healthcare resources to support primary care physicians in addressing the unique challenges of diabetes care in Egypt.

## Methods

An expert panel opinion was developed using a conference-based approach to discuss the current public health burden of T2DM in Egypt, the issue of managing patients with above-target glycemic levels, and the challenges encountered in current diabetes management practices. A panel of 13 professors of internal medicine and diabetology was invited, and all attended the conference. They were selected based on their recognized expertise, academic standing, and extensive clinical experience in diabetes management across Egypt. The experts were drawn from diverse universities and national institutes to ensure broad representation. Non-physician diabetes care providers (e.g., dietitians, diabetes care and education specialists) were not included in this expert panel opinion, as the primary objective was to develop simplified, physician-oriented guidance tailored to Egyptian primary care physicians. While no primary care physicians outside academia were directly included, the panel explicitly focused discussions on the barriers and challenges faced by primary care physicians in non-academic settings, drawing on national data and studies from primary care clinics.

Prior to the conference, the organizing committee identified and circulated a core set of international and regional guidelines (including the 2025 ADA Standards of Care in Diabetes, AHA cardiovascular guidelines, and Arab Diabetes Forum recommendations) [19, 20]. We also examined relevant AHA cardiovascular guidelines as they pertain to diabetes-related risk management [21, 22]. In addition, regionally tailored guidance, such as the Arab Diabetes Forum (ADF) clinical practice recommendations for the Arab region, was considered to capture context-specific practices [23]. As well as data from the literature pertaining to diabetes in Egypt and its management modalities [6, 11, 12, 17, 18, 24, 25]. Panelists were requested to review these documents in advance. During the expert panel opinion conference, these guidelines and selected literature were re-examined in a structured manner, with subgroup and plenary discussions focused on contextualizing their recommendations to the Egyptian primary care setting. Discussions centered around challenges faced by primary care physicians, barriers to effective management in primary care settings, and potential ways to improve diabetes management practice. The discussion was structured into thematic areas, including diagnosis, oral therapy, injectable therapy, cardiovascular disease, and kidney disease. Each subgroup conducted a focused review and presented draft recommendations.

Drafts were debated in plenary sessions, and recommendations were refined through structured discussion. Final decisions were made by unanimous agreement whenever possible, or by majority expert panel opinion after open discussion if differing viewpoints remained. The objective was to produce a simplified, context-relevant resource for Egyptian primary care physicians.

## Expert opinion and discussion

This expert panel opinion reflects the deliberations of a panel of 13 professors of internal medicine and diabetology drawn from seven major academic and national institutions across Egypt, including the University of Cairo, Alexandria, Ain Shams, Zagazig, Beni Suef, and Menoufia, and the National Institute of Diabetes and Endocrinology. This composition ensured balanced input from healthcare contexts.

For the structured discussion, the panel was divided into subgroups according to the major themes of this article: (a) treatment options and glycemic targets, (b) insulin regimens, (c) diabetes with cardiovascular disease, and (d) diabetic kidney disease. Each subgroup, building on its prior review of the circulated guidelines and literature, revisited these sources during the meeting to ensure a shared, thorough appraisal. International guidelines (ADA, AHA, Arab Diabetes Forum consensus) were systematically compared with Egyptian data and healthcare realities before draft recommendations were prepared. While being systematic and structured, this process did not employ a formal Delphi methodology. We recognize this as a limitation, but highlight that the chosen approach enabled the timely formulation of practical, Egypt-specific recommendations to guide primary care practice. Although the panel composition was limited to academic professors, the discussions were firmly anchored in the realities of everyday primary care practice in Egypt. Published data from rural and urban primary care clinics were used to guide the recommendations, ensuring that the guidance provided is relevant to non-academic settings.

### Treatment options and glycemic targets

A clear understanding of the complex pathophysiology of type 2 diabetes mellitus (T2DM) forms the foundation for therapeutic decision-making (Figure S1). The combined effects of insulin resistance, β-cell dysfunction, and excessive hepatic glucose output emphasize the importance of addressing multiple disease mechanisms. This insight supports the integration of lifestyle modification and pharmacologic therapies designed to target distinct pathways and improve clinical outcomes.

#### Oral antidiabetics

Oral anti-diabetic agents work through various mechanisms to regulate blood glucose levels and prevent complications associated with T2DM. These mechanisms involve improving insulin sensitivity in peripheral tissues, reducing glucose absorption, stimulating the pancreas to secrete more insulin, increasing the effectiveness of insulin in promoting glucose uptake and utilization by target tissues, and blocking glucose reabsorption in the kidneys [26, 27]. The main medication classes include metformin, sulfonylureas, non-sulfonylurea secretagogues, thiazolidinediones (TZDs), DPP-4 inhibitors, SGLT2 inhibitors, and GLP-1 receptor agonists. Although alpha-glucosidase inhibitors (AGIs) such as acarbose are available in Egypt, their use is relatively limited because of modest glycemic efficacy and gastrointestinal side effects; nevertheless, they remain a therapeutic option in selected patients. The choice of class (or combination of classes) is influenced by multiple clinical and non-clinical factors, including glycemic control, co-morbidities, patient preference, drug tolerability, cost, and affordability [26, 28, 29].

Importantly, drug selection should also consider the pathophysiological defects described by DeFronzo’s “egregious eleven” framework, which highlights the eleven key mechanisms contributing to hyperglycemia in T2DM. Targeting specific defects with appropriate drug classes allows a more rational and individualized approach to therapy.

Other important considerations include the presence of co-morbidities such as cardiovascular disease (CVD), kidney disease, liver disease, or heart failure (HF). In many cases, the presence of such co-morbidities not only restricts the use of certain agents due to contraindications but also actively supports the preferential use of specific medications with proven benefits in these settings (e.g., SGLT2 inhibitors for patients with kidney disease or heart failure, and GLP-1 receptor agonists for those with established cardiovascular disease). These co-morbidity-driven considerations are summarized in (Tables 1 and 2) [28, 30, 31].

**Table 1:** Oral hypoglycemic agents organ-centric profile

Drug group	Impact on ASCVD	Impact on HF	Impact on the kidneys	Impact on the liver	The target in the “egregious eleven”
Metformin	Potential Benefit	Neutral	Neutral (dose adjustment if eGFR < 45; discontinue if < 30)	Modest benefit in NAFLD/MAFLD; stop in advanced liver disease	Adipose tissue, Muscle, Liver, Colon/Microbiota
Sulfonylureas (Gliclazide, Glimepiride, Glibenclamide)	Neutral	Neutral	Neutral	Neutral	Beta cells
Non-SU Secretagogues (Repaglinide)	Neutral	Neutral	Neutral	Neutral	Beta cells
Thiazolidinediones (Pioglitazone, Rosiglitazone, TZDs)	Potential benefit (esp. stroke)	Increased risk	Neutral	Potential benefit in NAFLD/MAFLD	Adipose tissue, Muscle, Liver
DPP-4 Inhibitors (Sitagliptin, Vildagliptin, Linagliptin, Alogliptin)	Neutral	Neutral	Neutral	Neutral	Beta cells, Incretin effect, Alpha cells, Brain, Colon/Microbiota, Immune system
SGLT2 Inhibitors (Empagliflozin, Dapagliflozin)	Beneficial	Beneficial	Beneficial	Benefit in NAFLD/MAFLD	Kidney
GLP-1 Receptor Agonists (Oral Semaglutide)	Unknown (Benefit shown with SC formulations)	Neutral	Neutral	Potentially beneficial	Beta cells, Incretin effect, Alpha cells, Brain, Stomach/Small intestine
Alpha-glucosidase inhibitors (Acarbose, Miglitol)	Neutral	Neutral	Neutral	Neutral	Stomach/Small intestine

Targets in the “egregious eleven” correspond to pathophysiological defects in T2DM: Beta cells (insulin secretion), Incretin effect, Alpha cells (glucagon secretion), Adipose tissue, Muscle, Liver (hepatic glucose production), Brain (appetite/insulin resistance), Colon/Microbiota, Immune system/inflammation, Stomach/Small intestine (incretin/glucose absorption), and Kidney (glucose reabsorption). ASCVD, Atherosclerotic Cardiovascular Disease; HF, Heart Failure; eGFR, Estimated Glomerular Filtration Rate; NAFLD, Non-Alcoholic Fatty Liver Disease; MAFLD, Metabolic (Dysfunction)-Associated Fatty Liver Disease; SU, Sulfonylurea; TZDs, Thiazolidinediones; DPP-4, Dipeptidyl Peptidase-4; SGLT2, Sodium–Glucose Cotransporter-2; GLP-1, Glucagon-Like Peptide-1; SC, Subcutaneous;. cardiovascular benefit of GLP-1 RAs has been demonstrated only with subcutaneous formulations (e.g., liraglutide, semaglutide SC, dulaglutide), while evidence for oral GLP-1 RAs remains limited

**Table 2:** Oral hypoglycemic agents therapeutic profile

Drug class (examples)	Typical daily dose	eGFR guidance*	Efficacy	Efficacy in Combination Therapy	Increases Rates of Hypoglycemia	Impact on weight	Cost	Contraindications	Common adverse effects
Metformin	1–2 g/day in divided doses	Consider dose reduction if eGFR < 45 mL/min/1.73 m²; avoid if < 30 mL/min/1.73 m²	High	Yes	No	Neutral or mild loss	Low	Advanced renal disease; advanced liver disease	Gastrointestinal intolerance
Sulfonylureas (gliclazide, glimepiride, glibenclamide; micronized glibenclamide 1.25–12 mg)	Gliclazide 30–320 mg/day; glimepiride 1–8 mg/day; glibenclamide 1.25–20 mg/day	—	High	Yes	Moderate–high	Weight gain	Low	High risk of hypoglycemia (especially with glibenclamide); avoid in the elderly and in renal/hepatic impairment; advanced liver disease; moderate-to-severe renal impairment or renal failure.	—
Non-SU secretagogue Repaglinide	1.5–12 mg/day (in divided doses with meals)	No renal dose adjustment	Low–moderate	Yes	Low	Weight gain	Low	Advanced liver or kidney disease	—
Thiazolidinediones (TZDs; pioglitazone, rosiglitazone)	Pioglitazone 15–45 mg once daily; rosiglitazone 4–8 mg/day (1–2 doses)	No renal dose adjustment	High	Yes	No	Weight gain	Low	Incipient heart failure; advanced liver disease; chronic kidney disease with fluid retention risk; osteoporosis; severe anemia	Edema, fluid retention
DPP-4 inhibitors (sitagliptin, vildagliptin, linagliptin, alogliptin)	Sitagliptin 25–100 mg/day; vildagliptin 50–100 mg/day; linagliptin 5 mg/day; alogliptin 6.25–25 mg/day	Dose adjust in renal impairment (no adjustment for linagliptin)	Intermediate	Yes	No	Neutral	Intermediate	History of pancreatitis (caution)	—
SGLT2 inhibitors (empagliflozin, dapagliflozin)	Empagliflozin 10–25 mg once daily; dapagliflozin 5–10 mg once daily	Glycemic efficacy is limited when eGFR < 20 mL/min/1.73 m²	High	Yes	No	Weight loss	Intermediate	Conditions predisposing to DKA; peri-operative period; critical illness; prolonged fasting; significant volume depletion	Genital mycotic infections
GLP-1 receptor agonist (oral semaglutide)	Oral semaglutide 3 mg, 7 mg, or 14 mg once daily	No renal dose adjustment	High–very high	Yes	No	Marked weight loss	High	Personal or family history of medullary thyroid carcinoma; Multiple Endocrine Neoplasia, type 2; history of pancreatitis; gallbladder disease	Nausea, vomiting
Alpha-glucosidase inhibitors (acarbose, miglitol)	Acarbose 25–100 mg three times daily (TDS) with meals; miglitol 25–100 mg three times daily with meals	—	Low–moderate	Yes	Very low	Neutral or mild weight loss	Low–moderate	Advanced liver disease	Significant gastrointestinal intolerance (flatulence, diarrhea)

The eGFR values refer to mL/min/1.73 m². For agents with class-wide guidance, follow local labels for drug-specific renal dosing and monitoring. Where thresholds are provided (e.g., metformin < 45 for dose reduction; <30 to avoid; SGLT2 inhibitors < 20 with limited glycemic effect), interpret them within national labeling and patient context. MF metformin, GIT (GI) = gastrointestinal (tract), HF heart failure, GB gallbladder, CKD chronic kidney disease, DKA diabetic ketoacidosis, TDS three times daily. Sulfonylureas (e.g., gliclazide, glimepiride, glibenclamide) are widely available in Egypt. However, gliclazide and glimepiride are preferred due to their lower risk of hypoglycemia. Glibenclamide, a long-acting sulfonylurea, is associated with a significantly higher risk of hypoglycemia and should generally be avoided, particularly in older adults or patients with renal impairment. Its use should be restricted to circumstances where safer alternatives are not available

#### Non-insulin injectables

GLP-1 receptor agonists (GLP-1 RAs) are an important class of non-insulin injectable glucose-lowering therapies that deliver clinically meaningful HA1C reductions with low intrinsic risk of hypoglycemia and consistent weight loss, benefits that are particularly pertinent for people with T2D and overweight/obesity in primary care settings [32, 33]. Contemporary recommendations emphasize selecting agents that also address cardiovascular and kidney risk in patients with ASCVD, heart failure, or CKD, independent of baseline A1C or metformin use. Within this framework, GLP-1 RAs with established CV benefit (notably liraglutide, injectable semaglutide, and dulaglutide) are prioritized in patients with ASCVD or at high CV risk [20].

Renal outcome data have strengthened the positioning of this class. In FLOW, a randomized outcomes trial in adults with T2D and CKD, subcutaneous semaglutide significantly reduced the risk of clinically important kidney outcomes and death from cardiovascular causes versus placebo on top of standard care [34]. These findings support the use of GLP-1 RAs to complement or follow SGLT2 inhibitor therapy where additional kidney or cardiovascular protection is sought, or where SGLT2 inhibitors are contraindicated or not tolerated.

In Egypt, GLP-1 RAs are available and codified in the EDA Endocrine System Drug Formulary, which includes monographs for dulaglutide, exenatide (including extended-release), liraglutide, and semaglutide (injectable and oral). Conversely, amylin analogues (e.g., pramlintide) are not listed in the endocrine formulary, and their routine use in T2D care locally is not established [35]. Clinicians should therefore prioritize GLP-1 RAs when non-insulin injectable therapy is considered in Egyptian primary care.

#### Combination therapy

In current practice, initial therapy commonly begins with metformin; if targets are not achieved, an additional glucose-lowering agent with a complementary mechanism should be introduced [20]. In such cases, GLP-1 receptor agonists (injectable or oral) are integral options for dual or triple therapy (e.g., metformin + GLP-1 RA; SGLT2 inhibitor + GLP-1 RA; metformin + SGLT2 inhibitor + GLP-1 RA), given their robust A1C-lowering efficacy, favorable weight profile, and low intrinsic hypoglycemia risk [20]. Oral semaglutide offers an all-oral intensification pathway when injections are undesirable; dosing must follow fasting administration requirements. Combination of GLP-1 RAs with DPP-4 inhibitors should be avoided due to a lack of additional benefit. Where insulin is required, GLP-1 RA + basal insulin (free combination) or fixed-ratio combinations (insulin glargine/lixisenatide; insulin degludec/liraglutide) can improve glycemia with less weight gain and without increasing hypoglycemia versus basal insulin alone [36, 37].

Combination oral anti-diabetic therapy is recommended in the following cases: (a) A1C levels 1.5–2.0% above the target, (b) when more rapid achievement of glycemic goals is required [20]. Practically, physicians usually start with metformin as first-line therapy, usually for up to 6 months, to lower blood glucose levels. If the patient fails to reach the glycemic targets, an additional oral hypoglycemic agent (OHA) with a complementary mechanism of action should be introduced [28, 38]. Dual or triple OHA therapy or initiation of insulin may be required to achieve glucose targets more rapidly (≤ 3 months) and improve treatment adherence. The possible combination strategies are detailed in Table 3.

**Table 3:** Combination possibilities for therapy Intensifying*

Combination Therapy
Metformin + sulfonylureas
Metformin + DPP-4 inhibitors
Metformin + thiazolidinedione
Metformin + SGLT2 inhibitors
Metformin + Insulin + sulfonylureas
Metformin + Insulin + DPP-4 inhibitors
Metformin + GLP-1 receptor agonist
SGLT2 inhibitor + GLP-1 receptor agonist
Metformin + SGLT2 inhibitor + GLP-1 receptor agonist
Basal insulin + GLP-1 receptor agonist
Fixed-ratio basal insulin/GLP-1 receptor agonist

* Selection of combinations should be patient-centred (pathophysiology, comorbidities, hypoglycaemia risk, weight goals, access/cost, preference). If dual therapy is unlikely to meet targets or faster control is required, escalate to triple therapy (e.g., metformin + SGLT2 inhibitor + GLP-1 RA) or to insulin-containing regimens. Avoid combining a GLP-1 RA with a DPP-4 inhibitor (no added benefit). Oral semaglutide provides an all-oral intensification pathway but must follow fasting administration instructions. When introducing a GLP-1 RA with insulin or a sulfonylurea, consider dose reductions to mitigate hypoglycaemia. Basal insulin + GLP-1 RA (free or fixed-ratio) can improve glycaemia with less weight gain and without increasing hypoglycaemia versus basal insulin alone. SU, Sulfonylurea; DPP-4, Dipeptidyl Peptidase-4; TZDs, Thiazolidinediones; SGLT2, Sodium–Glucose Cotransporter-2; GLP-1, Glucagon-Like Peptide-1

#### Insulin therapy

In cases where oral and/or non-insulin injectable anti-diabetic agents alone or in combination are insufficient, insulin-based therapy might be considered as an add-on to the treatment regimen to provide an additional mechanism for controlling blood glucose levels [28, 39, 40]. Additionally, as T2DM progresses, oral and non-insulin injectable anti-diabetic agents may become less effective in maintaining blood glucose levels. Adding insulin therapy may help compensate for declining endogenous insulin production and provide the necessary insulin support [41].

Insulin is indicated for temporary intervention for individuals with T2DM in cases of:

1. Marked hyperglycemia (Fasting Plasma Glucose (FPG) > 250 mg/dL, HbA1c > 11%) with catabolic manifestations (e.g., unintentional weight loss, polyuria, polydipsia, or ketonuria) to overcome glucose toxicity and control glycemia,
2. Stressful situations, including but not limited to major surgery, severe infection, major trauma, acute coronary syndrome, hospitalization,
3. Insulin may also be indicated for patients on corticosteroid therapy,
4. Insulin should be considered for glycemic control during pregnancy.

The temporary intervention takes place until the condition is stabilized.

On the other hand, the permanent use of insulin is considered when:

1. Two or more oral or non-insulin injectable agents, in maximum tolerated doses, do not achieve the glucose-lowering targets.
2. In advanced kidney and liver diseases.

When it comes to initiating insulin therapy, several common fears and misconceptions may arise. These fears include anxiety around the use of needles or the requirement for multiple daily injections, concerns about experiencing hypoglycemia, and worries about potential weight gain. Additionally, several misconceptions about insulin therapy may impact the selection of a treatment regimen. These misconceptions include the belief that insulin is a last resort, a cause of complications, a sign of personal failure, and a negative influence on relationships and lifestyle [42, 43]. Patient education and psychological support are essential when starting insulin therapy to overcome barriers and correct misconceptions [42].

Insulin injection therapy aims to approximate the physiological insulin secretion pattern. The proposed insulin regimen options range from a single daily injection to multiple daily injections or the use of an insulin pump (Continuous Subcutaneous Insulin Infusion; CSII). More frequent insulin injections provide a closer approximation to natural insulin levels [44].

### Insulin regimen

#### Insulin initiation in T2DM patients

Injectable therapy should be considered in T2DM patients who have received two or more oral anti-diabetic drugs or non-insulin injectables and have not reached their individualized HbA1c target despite optimized therapy over approximately 3 months, considering adherence, appropriate dosing, and lifestyle interventions. This recommendation aligns with ADA’s guidance to reassess therapeutic regimens at least every 3 to 6 months when glycemic targets are unmet [20, 45]. Among patients with T2DM who do not achieve individualized HbA1c targets with oral agents, initiation of basal insulin is recommended as the first injectable therapy, a strategy well-established in clinical guidelines due to its efficacy, ease of use, and lower risk of hypoglycemia compared to more complex regimens [20, 46]. Basal insulin can be started at 10 units per day or 0.1–0.2 U/kg/day, based on body weight or fasting glucose levels [47].

**Fig. 1:**
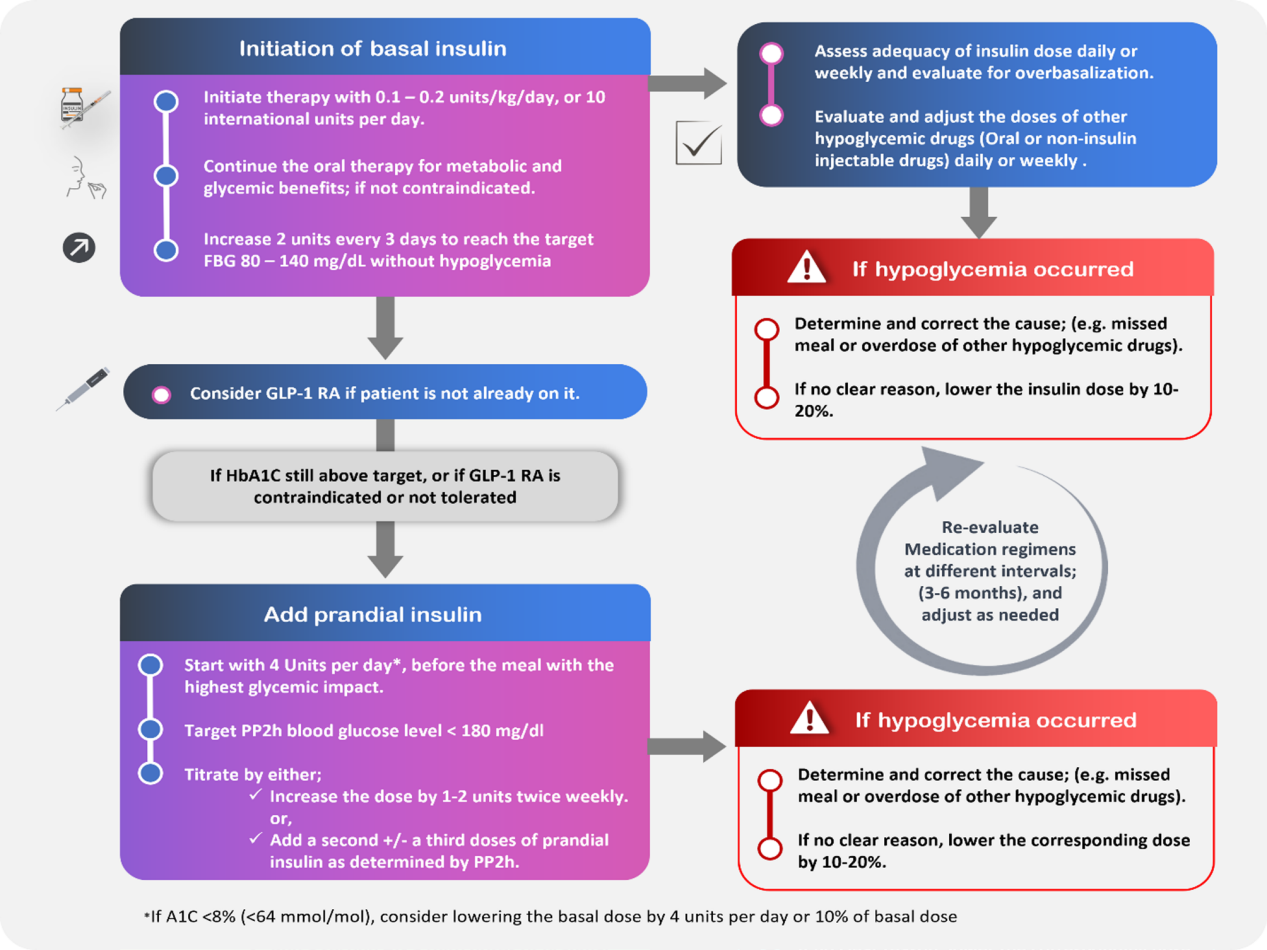
How to initiate and titrate insulin in people with T2DM. FBG, Fasting Blood Glucose; GLP-1 RA, Glucagon-Like Peptide-1 Receptor Agonist; HbA1c, Hemoglobin A1c; PP2h, Postprandial 2-Hour; A1C, Glycated Hemoglobin

Subsequent titration should be a gradual increase by 2–4 units or 10–15% once or twice weekly until the fasting glucose reaches the target range (80–130 mg/dL) [47]. If fasting glucose target was achieved but overall HbA1c remains elevated, primarily due to postprandial hyperglycemia, then stepwise insulin intensification is recommended, such as adding prandial insulin before the largest meal (basal-plus strategy), before consideration of full basal-bolus or premixed regimens [20, 47, 48]. While starting prandial insulin therapy, it is advised to continue using metformin, thiazolidinediones (TZDs), dipeptidyl peptidase-4 (DPP-4) inhibitors, and sodium-glucose cotransporter 2 (SGLT2) inhibitors [20]. Modifying or discontinuing sulfonylureas (SUs) is also essential to reduce the risk for hypoglycemia. Additionally, glucagon-like peptide-1 receptor agonists (GLP-1 RAs) can be utilized either as a free or fixed-dose combination with basal insulin [49] **(**Fig. 1**)**.

#### Insulin replacement therapy regimens in T2DM

In patients with T2D who fail to achieve glycemic targets despite optimization of basal insulin, structured intensification of therapy becomes necessary. The first step in this escalation is the basal-plus approach, whereby a single prandial insulin injection, typically administered before the meal with the greatest carbohydrate content, is added to the existing basal regimen [50, 51]. If hyperglycemia persists, progression to a full basal-bolus regimen or premixed insulin schedule may then be warranted. This stepwise intensification strategy is supported by guidelines and aims to balance glycemic efficacy with simplicity and patient tolerability [52].

Evidence indicates that initiating a prandial injection as part of a basal-plus strategy allows significant improvement in postprandial glycemic control with minimal increase in regimen complexity. This approach is often preferred before moving to full basal-bolus schedules [50]. When necessary, full basal-bolus regimens can be initiated, consisting of basal insulin supplemented with rapid-acting insulin at each meal to mimic physiological insulin patterns and achieve tighter glycemic control [53]. Alternatively, premixed insulin formulations (e.g., 70/30 or 75/25 mixtures) may be used in settings where simpler regimens are preferred or multiple daily injections are not feasible [54].

##### Pre-mixed insulin regimen

Pre-mixed insulin formulations combine Neutral Protamine Hagedorn (NPH) as intermediate-acting insulin with either regular human insulin or rapid-acting human insulin analogues, providing bolus insulin coverage for meal-time glucose control and basal insulin coverage for background glucose regulation [44, 55]. Pre-mixed insulin is typically recommended for patients with regular meal timing and activity routines. In Egypt, the common ratio is 70% NPH and 30% Regular insulin. It is available in vials, pens, and pre-filled cartridges. Pre-mixed insulin offers advantages such as reduced injections and cost-effectiveness. However, challenges include NPH-induced glucose variability and the need for a fixed daily schedule, which can be mitigated through careful planning of meals and activities. The pre-mixed insulin regimen initiation process involves several steps. First, the total daily dose in international units (TDD) is calculated by multiplying body weight in kilograms by 0.3 to 0.5 international units (IU). Next, the TDD is divided into two-thirds before breakfast and one-third before dinner, or equal halves if the evening meal is the main meal. Dose titration should occur weekly based on the lowest self-monitored plasma glucose (SMPG) value from the previous three days, with the morning dose adjusted according to pre-dinner blood glucose values and the evening dose according to FPG values [28].

Adjust pre-mixed insulin doses based on blood glucose values as follows [56]:

- Less than 80 mg/dL: decrease by 2 units.
- 80–130 mg/dL: no change.
- 131–150 mg/dL: increase by 2 units.
- 151–180 mg/dL: increase by 4 units.
- Greater than 180 mg/dL: increase by 6 units.

Human pre-mixed insulin can be injected 30 min before meals, but pre-mixed analogues can be injected immediately before meals [56]. Dose titration involves starting with a low dose and gradually increasing it until the target is reached, while dose adjustment means increasing or decreasing the dose to maintain the level at the target [47, 57–59]. When titrating the morning dose, checking the pre-lunch blood glucose level is advisable. If it is less than 130 mg/dL and the pre-dinner blood glucose level is still not at the target, adding a third injection of pre-mixed insulin before lunch should be considered, starting with 6 units and titrating weekly based on pre-dinner blood glucose levels [47]. Patients on the pre-mixed regimen should follow a meal plan that includes three main meals and three snacks in between to protect against hypoglycemia before meals and overnight [60].

##### Basal-bolus insulin regimen

The basal-bolus insulin and basal-plus regimens combine long-acting basal insulin and rapid-acting insulin at mealtimes. Full basal-bolus therapy consists of basal insulin and three daily injections of short-acting insulin at mealtime, while the term “basal-plus therapy” refers to a regimen that includes a basal insulin injection, with the gradual addition of one to three pre-prandial short-acting insulin doses per day. These regimens are designed to mimic the physiological insulin release from the pancreas [44], with the long-acting insulin keeping blood glucose levels stable during periods of fasting and the rapid-acting insulin acting on blood glucose levels resulting from meals. The basal-bolus and basal-plus regimens allow for more flexible management of blood glucose levels, tailored to individual scheduling of meals [61–63]. They are recommended for individuals with variable daily routines who are willing to self-monitor their blood glucose levels and adjust their doses accordingly. While these regimens effectively mimic natural insulin release and provide flexibility in meal timing and carbohydrate intake, they also require multiple daily injections and frequent monitoring, making them unsuitable for some patients [44, 62].

The initiation of a basal-bolus insulin regimen involves calculating the total daily dose (TDD) of insulin (Figure S2), dividing it into 50% for basal insulin and 50% for bolus insulin, and then dividing the bolus insulin dose into thirds for breakfast, lunch, and dinner [28].

When titrating basal insulin in a basal-bolus insulin regimen, the dose should be adjusted based on FPG levels [47, 57, 58]. The recommended titration strategy involves increasing the dose by 2 units every 3 days until FPG levels are within the target range of 80–130 mg/dL [59, 64]. If FPG levels exceed 180 mg/dL, the dose is increased by 4 units every 3 days. If FPG levels drop below 80 mg/dL, the dose is decreased by 2 units, and if they fall below 70 mg/dL, the dose is decreased by 4 units.

For bolus insulin titration, adjustments are guided by postprandial glucose (PPG) monitoring. A practical approach is to increase the bolus dose by 1–2 units every 3 days until PPG levels fall within the target range of 140–180 mg/dL. Larger dose adjustments may be considered in patients with persistently elevated PPG values despite multiple small increments, particularly in those with higher baseline insulin requirements. Conversely, if PPG values fall below 140 mg/dL, the bolus dose should be reduced to minimize hypoglycemia risk [59, 64]. The insulin therapy initiation always varies according to individual preferences and adherence to the therapy [65]; therefore, one regimen may be preferred over others, considering the factors in Table 4.

**Table 4:** Determinants for choosing an insulin intensification regimen [66]

Factor	Premixed insulin	Basal–bolus therapy
Injection frequency preference	Suitable for patients preferring fewer injections	Suitable for patients who are comfortable with multiple daily injections
Self-monitoring of blood glucose (SMBG)	Suitable for patients who are reluctant to self-monitor more than twice daily	Suitable for patients who are willing to self-monitor at least 3–4 times daily
Lifestyle consistency, including meal timing and carbohydrate intake	Suitable for patients with a consistent daily routine	Suitable for patients with a variable daily routine
Postprandial hyperglycemia management	More suitable for those with minimal postprandial hyperglycemia	More suitable for those with significant postprandial hyperglycemia
Ability to adhere to the regimen	May be suitable for patients with limited cognitive function or lower treatment flexibility	Recommended for motivated patients with good cognitive function and the capacity to manage complex regimens
Availability of educational and emotional support	May be considered for patients with limited support systems	Most appropriate for patients with access to structured educational and emotional support

*SMBG* Self-Monitoring of Blood Glucose

### Diabetes with cardiovascular disease

Cardiovascular disease (CVD) is a prevalent comorbidity in patients with diabetes, where T2DM patients are subject to a two- to three-fold increase in the risk of developing CVD, with a reported co-prevalence exceeding 30% [67, 68]. Correspondingly, CVD is the leading cause of death among individuals with T2DM [68], with CV mortality rates being drastically higher than those in non-diabetics [69], with the estimated rates being higher in low- and middle-income countries [70]. The increased risk of CVD in T2DM can be attributed to insulin resistance, vascular inflammation, and increased oxidative stress; these pathological trends result in increased vascular damage and atherosclerosis [69, 71]. Both T2DM and CVD share common risk factors, such as obesity, hypertension, and dyslipidemia, which further enhances the codependent relationship between both conditions [71]. Consequently, management plans for T2DM presenting with CVD co-morbidities should be tailored for each patient’s needs using a stepwise approach that involves risk assessment, setting targets for glycemic control, choice of anti-diabetic agents, and risk factors management.

#### Diagnosis and risk assessment

Given the great health and economic burden of CVD and its manifestations, assessing the risk for CVD in newly diagnosed T2DM patients is essential. In 2021, the European Society of Cardiology (ESC) proposed a CVD risk prediction model, SCORE2, to identify individuals at risk of CVD and guide clinical decisions for these individuals [72], which was recently updated to SCORE2-Diabetes, taking into account geographical variations of CVD risk associated with T2DM [71, 72]. The SCORE2-Diabetes algorithm allows for the categorization of T2DM into four groups according to the calculated score, presence of atherosclerotic CVD (ASCVD), and evidence of target-organ damage (TOD**).**

#### Management

##### Determination of glycemic targets

Glycemic targets for T2DM patients with CVD are determined on an individual basis, where the CV benefits of tight glycemic control with an HbA1c target of 7% or less are limited to long-term ones. Conversely, hypoglycemia poses a heightened risk for people with concomitant vascular damage. Therefore, in individuals with shorter life expectancy or those at higher risk of hypoglycemia, less stringent HbA1c targets may be appropriate to minimize treatment burden and avoid hypoglycemia. In contrast, in individuals with longer life expectancy and lower risk of hypoglycemia, more stringent glycemic targets may be pursued to reduce the long-term risk of diabetes-related complications (Figure S3) [5, 70]. Managing glucose control in diabetic patients with high CV risk requires addressing several glycemic measures, such as personalizing HbA1c targets, reducing hypoglycemic episodes, and minimizing glucose variability [73].

##### Choice of CV-risk-based anti-diabetic agent

T2DM individuals with “high” or “very high” CVD risk should receive anti-diabetic agents with proven CV benefits (GLP-1 RAs and SGLT-2 inhibitors) regardless of their glycemic control status. If needed to achieve glycemic goals, use of anti-diabetic agents showing potential CV benefits (e.g., metformin, pioglitazone) or proven CV safety (e.g., DPP-4 inhibitors, lixisenatide, exenatide, insulin, glimepiride) with lower risks of hypoglycemia is recommended [49]. Insulin and sulfonylureas should not be regarded as CV-risk–based therapeutic choices due to their higher risk of hypoglycemia, although they may still be required for glycemic control when other options are insufficient or unavailable. The SCORE2-Diabetes algorithm summarizes the risk assessment for T2DM patients. Risk categorization is determined based on the presence of atherosclerotic cardiovascular disease or severe TOD [71] (Table S1 and S2).

##### Management of risk factors

- Hypertension (Figure S4)
  Hypertension is a major modifiable CVD risk factor, and its concurrent existence with T2DM leads to an additive and sometimes synergetic effect on CVD risk. The majority of diabetic patients have associated hypertension with a prevalence of 80% and 87% in males and females, respectively [73, 74]. Consequently, regular blood pressure (BP) measurement and monitoring, considering standardized conditions for each measurement, are essential for people with T2DM starting at their initial diagnosis at every clinical visit [75]. Additionally, a value of 180/110 mmHg or more in individuals with CVD is reasonable for diagnosing hypertension at a single visit [76]. Blood pressure control reduces stroke, coronary events, and kidney disease risks [49].
  Although no nationally representative data are currently available on the prevalence of hypertension among people with diabetes in Egypt, global evidence consistently shows that more than half of patients with T2DM develop hypertension [77]. This highlights the importance of integrated management in Egyptian primary care, with early detection and blood pressure control prioritized to reduce cardiovascular and renal complications. Our expert panel opinion, therefore, emphasizes screening at every visit and the use of renin–angiotensin system blockers (ACE inhibitors or ARBs) as first-line agents when hypertension coexists with diabetes. The “100 million seha” (100 million healthy lives) initiative in Egypt was launched to detect and manage diabetes mellitus and hypertension early among adults through nationwide free screening programs. Building on this effort, the initiative has significantly expanded access to preventive care, lifestyle counseling, and treatment services across the country.
**What are the treatment targets?**
  Pharmacological intervention is recommended for diabetic patients with blood pressure measurement of > 130 mmHg, with a systolic blood pressure target of 129 mmHg or less to reduce CVD risk. However, in patients aged 85 years and over, the ADA guidelines recommend a target of 130/80 for the healthy ones and those with few complications and 140/90 for those with very complex conditions, or bad health status [78].
**How to manage and evaluate?**
  A combination therapy of a renin-angiotensin system (RAS) inhibitor and either a calcium channel blocker (CCB) or a thiazide/thiazide-like diuretic is recommended in these cases [49].
  Regarding Lifestyle: The recommendations for diabetic patients comorbid with hypertension involve weight loss, if overweight, physical activity, alcohol restriction, reduction of sodium intake, in addition to a balanced diet, low in high-fat dairy products and rich in vegetables and fruits [79].
  Medical intervention: Combination therapy using a RAS inhibitor Angiotensin-Converting Enzyme Inhibitor (ACE-I), Angiotensin II Receptor Blocker (ARB), and either a CCB or a thiazide/thiazide-like diuretic is considered as a starting point [49].
  Monitoring: Home blood pressure self-monitoring (HBPM) should be considered for people with diabetes on antihypertensive treatments [80]. Additionally, 24-hour ambulatory BP monitoring can assess abnormal 24-hour BP patterns, including nocturnal hypertension and altered nocturnal BP dipping, to adjust antihypertensive treatment [81].
- Dyslipidemia
  Dyslipidemia is one of the common complications of T2DM as a result of insulin resistance [82]; it also poses a major risk factor for CVD, and therefore, lipid targets are determined in accordance with CV risk assessment. Pharmacological treatment options include statins as a first-line therapy, with ezetimibe and Proprotein Convertase Subtilisin/Kexin Type 9 (PCSK9) inhibitors as add-on therapy options in case of persistent dyslipidemia. Ezetimibe and PCSK9 inhibitors can also be used when statin-based therapy is not tolerated. Moreover, high-dose icosapent ethyl should be used in combination with statins in patients presenting with hypertriglyceridemia [49].
  In patients with severe hypertriglyceridemia (≥ 500 mg/dL), fibrates in combination with omega-3 are recommended to reduce the risk of pancreatitis [83]. Fibrates may also be considered in combination with statins for patients with persistently high triglycerides and low HDL-C, although evidence for cardiovascular benefit remains limited [84].
  In adults with diabetes, it is reasonable to obtain a lipid profile (total cholesterol, LDL cholesterol, HDL cholesterol, and triglycerides) at the time of diagnosis, at the initial medical evaluation, and at least every 5 years thereafter in individuals < 40 years of age. In younger people with longer duration of disease (such as those with youth-onset type 1 diabetes), more frequent lipid profiles may be reasonable. A lipid panel should also be obtained immediately before initiating statin therapy. Once an individual is taking a statin, LDL cholesterol levels should be assessed 4–12 weeks after initiation of statin therapy, after any change in dose, and annually (e.g., to monitor for medication taking and efficacy). Monitoring lipid profiles after initiation of statin therapy and during therapy increases dose titration and statin adherence [85, 86].
**Lipid-Lowering treatment**
  Lipid-lowering treatment is crucial for diabetes management, particularly in patients with above-target Low-Density Lipoprotein Cholesterol (LDL-C) levels. Statins are the first choice for lowering LDL-C in individuals with diabetes and elevated LDL-C levels [87, 88]. If the target LDL-C is not reached with statin therapy, adding ezetimibe should be considered [89]. For patients with very high cardiovascular risk who have persistently elevated LDL-C levels despite taking the maximum tolerated doses of statins combined with ezetimibe, or for those who are intolerant to statins, the use of PCSK9 inhibitors should be considered [90]. If a statin-based regimen is not tolerated at any dose, ezetimibe can be combined with a PCSK9 inhibitor or used alone. Additionally, high-dose icosapent ethyl (2 g twice daily) may be considered alongside a statin for patients with hypertriglyceridemia, as shown in Table S3 [49].
- Heart Failure
  Heart failure (HF) is a prevalent manifestation of CVD presenting concurrently with T2DM. Both DM and HF are considered major risk factors for each other [70, 91]. Studies have reported a 2- to 4-fold increase in HF prevalence in diabetic patients, correlating this to vascular changes caused by hyperglycemia and hyperinsulinemia [92]. HF can present with preserved ejection fraction (HFpEF), mildly reduced ejection fraction (HFmrEF), or reduced ejection fraction (HFrEF) [43]. To date, reports on prevalence rates of these phenotypes in people with diabetes do not differ from those in nondiabetics; however, the increased risk of worsening HF outcomes associated with diabetes is more prominent in the case of HFrEF [93].
  To facilitate early detection, it is recommended to systematically survey for HF symptoms and signs at each clinical encounter, particularly in individuals with established atherosclerotic cardiovascular disease, hypertension, or chronic kidney disease [94, 95]. Additionally, a natriuretic peptide (BNP or NT-proBNP) measurement should be considered for screening asymptomatic patients at risk for heart failure. The American Diabetes Association’s 2025 Standards of Care recommend considering natriuretic peptide screening to facilitate the prevention of symptomatic heart failure [96]. A positive screening result (e.g., NT-proBNP ≥ 125 pg/mL) should prompt further evaluation with echocardiography to identify structural heart disease [96]. A consensus report by some European societies suggests routine evaluation every two to three years for low-risk patients and annually for high-risk patients when natriuretic peptide levels are below 125 pg/mL and in the absence of suspected heart disease [95].

##### Screening and diagnosis

To facilitate early detection of HF in diabetic patients, it is recommended to systematically survey HF symptoms and/or signs at each clinical encounter for individuals with diabetes [90].

A diagnostic algorithm is then followed in case HF is suspected. In case of suspecting an HF patient, measuring B-type natriuretic peptide and N-terminal pro-B-type natriuretic peptide (Class I, Level B) is recommended by the ESC guidelines [45]. The following diagnostic tests should be conducted for all patients suspected of having heart failure (Class I, Level C) [49, 70]: a 12-lead ECG, transthoracic echocardiography, chest radiography (X-ray), and routine blood tests to identify co-morbidities, including Complete Blood Count (CBC), creatinine, urea, thyroid function tests, and electrolytes. 

##### Management and recommendations

Management recommendations of HF in T2DM patients elaborate on glucose-lowering medications use with evidence of a good safety profile and add-on agents for additional glycemic control [49]. SGLT2 inhibitors are indicated in T2DM patients, comorbid with HF, for minimizing HF hospitalization and CV death risks [97]. Add-on glucose-lowering agents may be used for additional control, but only if those agents’ effect on the HF hospitalization risk is neutral. However, Pioglitazone and Saxagliptin are not recommended as Glucose-lowering agents since they are linked to an increased HF hospitalization risk [49, 98, 99].

### Diabetic kidney disease

Renal complications represent a great burden in T2DM patients, with 20% to 40% developing diabetic kidney disease (DKD) [100]. DKD is a chronic progressive condition that represents the leading cause of end-stage renal disease (ESRD) and renal replacement therapy worldwide, where it accounts for half of ESRD cases in the United States [101, 102]. Correspondingly, DKD was reported to be Egypt’s second most common cause of ESRD [25].

The pathogenesis of DKD is predominantly rooted in hyperglycemia, resulting in reduced estimated glomerular filtration rate (eGFR) and, in many cases, albuminuria; however, multiple pathogenic pathways contribute to this progression [103]. Consequently, DKD can manifest in individuals with T2DM with or without proteinuria or diabetic retinopathy [104]. Given its complex nature, effective management often requires a multifactorial approach, incorporating glycemic control, blood pressure management, renin-angiotensin-aldosterone system (RAAS) blockade, and SGLT2 inhibitors to mitigate multiple pathophysiological disturbances simultaneously [103]. The decline in renal function is typically observed in patients with T1DM after about 10 years of history with diabetes; in contrast, T2DM patients often present with DKD at the time of their first diagnosis, as both conditions tend to go unnoticed for long periods [105].

### Screening and management of diabetes-associated complications

In its early stages, DKD is asymptomatic and can only be detected by measuring the decline in kidney function (i.e., eGFR) and albuminuria levels; hence, the 2025 ADA Standards of Care recommend the regular assessment of eGFR and spot urinary albumin-to-creatinine ratio (spot UACR) in both T1DM patients (after five years of their initial DM diagnosis and annually thereafter) and T2DM patients (at the time of their first diagnosis and annually thereafter). A diagnosis of DKD is then made if either a low eGFR or persistent albuminuria is detected, with the latter being defined as 2 or more abnormal UACR results collected over 3 to 6 months [106]. If a DKD diagnosis is established, DKD patients should be monitored regularly according to the disease stage. Additionally, DKD patients should be regularly monitored for signs and symptoms of CKD complications, including volume overload, electrolyte imbalance, anemia, and metabolic bone disorders [107].

#### Management and screening

Pharmacological management of DKD aims to maintain kidney function and slow DKD’s progression into ESRD. It involves a multidisciplinary approach that includes glucose-lowering (Figure S5), RAAS inhibition, SGLT-2 inhibition, and albuminuria reduction [106–108].

For patients with diabetic kidney disease (DKD), the typical glycemic target is HbA1c **<** 7%. Metformin is the first-line glucose-lowering agent unless the patient has a moderate to severe decline in eGFR. It should be discontinued if eGFR falls below 30 mL/min/1.73 m² and should not be initiated if eGFR is below 45 mL/min/1.73 m² [109]. SGLT2 inhibitors are recommended for all T2DM patients with DKD, irrespective of glycemic control, due to their nephroprotective and cardiovascular benefits, even at eGFR levels as low as 25 mL/min/1.73 m² [110]. If glucose levels remain uncontrolled with metformin and SGLT2 inhibitors, adding a GLP-1 receptor agonist is recommended (Figure S5) [107, 111].

Reducing albuminuria is recommended for patients with albuminuria levels of ≥ 30 mg/day [112]. Finerenone, a nonsteroidal selective mineralocorticoid receptor antagonist, is recommended at a daily dose of 20 mg for patients with eGFR ≥ 60 mL/min/1.73 m² and 10 mg for those with eGFR between 25 and 60 mL/min/1.73 m². Serum potassium and creatinine levels should be measured four weeks after starting therapy [113]. ACE inhibitors or ARBs are first-line in diabetes with hypertension and albuminuria because they lower intraglomerular pressure and reduce albuminuria [106, 114]. SGLT2 inhibitors are recommended for people with type 2 diabetes and chronic kidney disease to slow CKD progression and reduce albuminuria, benefits that extend beyond glucose lowering [106, 115, 116]. GLP-1 receptor agonists may be considered when additional cardio-renal risk reduction is desired; ADA 2025 notes kidney benefits (including reductions in albuminuria) as part of broader outcome data, particularly when SGLT2 inhibitors are contraindicated or insufficient [19, 117, 118]. Management plans should be tailored to individual risk factors and coexisting conditions, with modifications as necessary.

##### Diabetic retinopathy 

Diabetic retinopathy is a leading cause of preventable vision loss in adults with type 2 diabetes, with risk strongly tied to disease duration, glycemic control, hypertension, dyslipidemia, and presence of nephropathy [119]. The ADA 2025 Standards recommend an initial dilated, comprehensive eye examination at the time of T2DM diagnosis, followed by annual screening or every 1–2 years if prior exams are normal and glycemia is well-controlled. Maintaining near-normoglycemic levels and optimizing blood pressure and lipid control have been shown to prevent or slow the progression of retinopathy and reduce the need for ocular surgery. When retinopathy is detected, prompt referral to ophthalmology is essential. Advanced disease stages such as proliferative retinopathy or diabetic macular edema should be managed with timely interventions like pan-retinal photocoagulation or intravitreal anti-VEGF therapy [119].

##### Peripheral neuropathy

Peripheral diabetic neuropathy is one of the most prevalent complications of diabetes, affecting up to 50% of patients over time and contributing to foot ulcers, Charcot deformities, and increased fall risk [120]. The ADA recommends annual comprehensive foot exams beginning at diagnosis of type 2 diabetes, including visual inspection, 10-g monofilament testing, and assessment of temperature or vibration sensation; for type 1 diabetes, screening starts five years after diagnosis. Prevention hinges on tight glycemic control, healthy lifestyle (diet, exercise, smoking cessation), and patient education on foot hygiene and self-examination [121]. There is no known cure for neuropathy, so management focuses on preserving foot integrity and quality of life using pharmacologic agents (e.g., duloxetine, pregabalin) for symptomatic relief and ensuring protective footwear or podiatry interventions where available. 

##### Peripheral arterial disease (PAD)

Peripheral arterial disease is frequently underdiagnosed in type 2 diabetes, yet carries significant risks for cardiovascular events and limb complications [122]. Major societies, including the ESC, AHA, and ADA, recommend screening with the ankle–brachial index (ABI) in individuals aged ≥ 50 years, or those with long-standing diabetes, smoking, hypertension, dyslipidemia, or existing microvascular disease [122, 123]. The ABI is a non-invasive, sensitive test (≈ 90%) with high specificity (≈ 98%) for identifying PAD. Early detection allows initiation of preventive strategies such as smoking cessation, supervised exercise programs, lipid-lowering and antiplatelet therapy, and blood pressure optimization measures shown to reduce both cardiovascular risk and limb complications [124]. Patients with critical limb ischemia or claudication should be referred to vascular specialists for advanced diagnostics and potential revascularization. 

##### Uncontrolled hypertension

Hypertension can negatively affect DKD progression, and accordingly, patients should be treated to a target of < 130/80 mmHg. A combination of RAS inhibition and calcium-channel blocking (dihydropyridine class) is optimal in most cases. However, close monitoring of serum creatinine and potassium levels is recommended. A combination of an ACE-I with an ARB is not recommended due to a lack of evidence for additional cardiovascular benefits and an increased risk of adverse events, including hyperkalemia, hypotension, and acute kidney injury [125–128]. 

##### Cardiovascular complications

Chronic kidney disease coexistence with diabetes drastically increases the risk for cardiovascular complications, where about half of the patients with advanced DKD develop cardiovascular diseases and are at high risk of mortality due to cardiovascular complications [129]. Consequently, statins as an additive therapy are highly recommended for DKD patients, preferably atorvastatin or fluvastatin, because both do not require dose adjustment with the decline in eGFR [107, 130]. Figure S6 outlines the algorithm for preventing DKD progression. 

##### Non-pharmacological management

Lifestyle modifications are essential to any DKD management strategy. Recommended modifications include regular exercise, weight loss if overweight/obese, and dietary adjustments [131]. Achieving even modest weight loss in overweight or obese individuals with T2DM yields measurable clinical benefits. The 2025 ADA Standards of Care recommend targeting an initial weight loss of 3–7% of baseline body weight, achieved through a structured, multi-component lifestyle intervention comprising nutrition, physical activity, and behavior therapy. For some patients, especially those aiming for diabetes remission, a more ambitious up to 15% weight loss may be appropriate. To preserve muscle mass, it is advised that weight-loss efforts include muscle-strengthening exercises alongside aerobic activity [132].

To create the necessary energy deficit, patients are encouraged to target a daily reduction of 500–750 kcal, typically achieved through an eating plan of 1,200–1,500 kcal/day for women and 1,500–1,800 kcal/day for men [133]. Even a 5% reduction in body weight can significantly improve glycemia, blood pressure, lipid profiles, and long-term cardiovascular risk, with incremental benefits as more weight is lost [133]. Furthermore, structuring weight-loss support using an interprofessional team including dietitians, diabetes educators, and behavioral counselors increases the effectiveness of lifestyle interventions [134]. 

##### Referral to a nephrologist

Prompt referral for DKD—or suspected DKD—is advised for individuals with (a) uncertain etiology or atypical clinical features suggesting a possible non-diabetic cause of kidney disease (e.g., rapid eGFR decline, hematuria, active urinary sediment, or absence of diabetic retinopathy), (b) advanced CKD complications (Table S4), (c) an eGFR < 30 mL/min/1.73 m², or (d) who may be candidates for renal replacement therapy [135].

## Limitations

A key limitation of this work is that the panel consisted exclusively of academic professors, without direct participation of non-professor primary care physicians. Consequently, the recommendations may not fully capture the lived experience of frontline providers in non-academic settings. However, the panel reviewed evidence from Egyptian primary care clinics and explicitly discussed barriers in these environments, which helped ensure practical relevance.

## Conclusions

This expert panel opinion translates global evidence into practical, locally relevant recommendations for the management of T2DM in Egyptian primary care settings. The panel emphasizes individualized glycemic targets, stepwise initiation and intensification of therapy, and integration of cardiovascular- and kidney-protective agents where appropriate. Attention to patient education, adherence, and continuity of care is critical to improving outcomes. Importantly, understanding the multifactorial pathophysiology of T2DM provides the scientific rationale for these therapeutic strategies. Moving forward, efforts should focus on closing the gap between guidelines and clinical practice, improving access to essential therapies, and supporting primary care physicians in delivering comprehensive and patient-centered diabetes care.

## Recommendations

Primary care physicians’ contribution to diabetic patients’ care involves management of glycemia to the appropriate target for each patient, screening for diabetes-associated complications, and intervening early to mitigate complications. Healthcare practitioners are encouraged to adopt tailor-made plans for the patients, such as changes in lifestyle habits and self-blood glucose monitoring, to ensure effectiveness in patient management outcomes. Review of medication and change of dosage ought to be undertaken routinely as necessary to ensure that treatment remains effective and tolerable. Also, treatment adherence can be supported with more aggressive treatment of diabetes through the inclusion of diabetes educators, dietitians, and pharmacists in the patient’s care.

## Supplementary Information

Supplementary Material 1.

## Data Availability

Not applicable. This article does not report new datasets or primary research data.

## Abbreviations

ADA: American Diabetes Association
ADVANCE: Action in Diabetes and Vascular Disease: Preterax and Diamicron Modified Release Controlled Evaluation
AHA: American Heart Association
ASCVD: Atherosclerotic Cardiovascular Disease
A1C/HbA1c: Hemoglobin A1c (Glycated Hemoglobin)
ARB: Angiotensin II Receptor Blocker
ACE-I: Angiotensin-Converting Enzyme Inhibitor
BNP: B-type Natriuretic Peptide
CBC: Complete Blood Count
CKD: Chronic Kidney Disease
CVD: Cardiovascular Disease
DKA: Diabetic Ketoacidosis
DKD: Diabetic Kidney Disease
DM: Diabetes Mellitus
DPP-4: Dipeptidyl Peptidase-4
eGFR: Estimated Glomerular Filtration Rate
ESC: European Society of Cardiology
ESRD: End-Stage Renal Disease
FPG: Fasting Plasma Glucose
GB: Gallbladder
GLP-1 RAs: Glucagon-Like Peptide-1 Receptor Agonists
GIT: Gastrointestinal Tract
HCP: Healthcare Provider
HF: Heart Failure
HFmrEF: Heart Failure with Mildly Reduced Ejection Fraction
HFpEF: Heart Failure with Preserved Ejection Fraction
HFrEF: Heart Failure with Reduced Ejection Fraction
IDF: International Diabetes Federation
IU: International Unit
LDL-C: Low-Density Lipoprotein Cholesterol
MAFLD: Metabolic Dysfunction-Associated Fatty Liver Disease
NAFLD: Non-Alcoholic Fatty Liver Disease
NPH: Neutral Protamine Hagedorn
OHA: Oral Hypoglycemic Agent
PCSK9: Proprotein Convertase Subtilisin/Kexin Type 9
PPG: Postprandial Glucose
RAAS: Renin-Angiotensin-Aldosterone System
SCORE2: Systematic Coronary Risk Estimation 2
SMPG: Self-Monitored Plasma Glucose
SGLT2: Sodium-Glucose Cotransporter-2
SU: Sulfonylurea
T1DM: Type 1 Diabetes Mellitus
T2DM: Type 2 Diabetes Mellitus
TDD: Total Daily Dose
TOD: Target-Organ Damage
TZDs: Thiazolidinediones
UACR: Urinary Albumin-to-Creatinine Ratio
