# Supplementary material for: From guidelines to practice: an Egyptian expert opinion on type 2 diabetes mellitus management in primary care settings
Source: BMC Prim Care. 2025 Nov 27;26:392. doi: 10.1186/s12875-025-03091-7 (PMC12687541; doi:10.1186/s12875-025-03091-7)
Supplement: Supplementary file 1 — Supplementary Material 1. [file 12875_2025_3091_MOESM1_ESM.docx]

**SUPPLEMENTARY DATA**

**From Guidelines to Practice: Type 2 Diabetes Management in Primary Care Settings**

| Tables | Page Number |
| --- | --- |
| Table S1 - SCORE2-DIABETES for Men | 4 |
| Table S2 - SCORE2-DIABETES for Women | 6 |
| Table S3. Lipid-lowering medications | 8 |
| Table S4. Key clinical and laboratory evaluations for chronic kidney disease complications in T2DM | 8 |

| Figures | Page |
| --- | --- |
| Figure S1. Type 2 diabetes pathophysiology | 9 |
| Figure S2. Illustration of targeted daily dose calculation of basal-bolus insulin regimen. | 10 |
| Figure S3. Simplified Guide to Glycemic Targets in Type 2 Diabetes with Cardiovascular Disease. | 11 |
| Figure S4. Screening and diagnosis of hypertension in patients with diabetes. | 12 |
| Figure S5. Glycemic management for people with diabetes and DKD. | 13 |
| Figure S6. Algorithm for prevention of diabetic kidney disease progression. | 14 |

# **Table S1:** SCORE2-DIABETES for Men (1)

| **Points table for men with diabetes and current age 40-69** | | | | | | | |  | **Reported points by age column** |
| --- | --- | --- | --- | --- | --- | --- | --- | --- | --- |
| **Risk predictor** | **Risk predictor category** | **Age 40-44** | **Age 45-49** | **Age 50-54** | **Age 55-59** | **Age 60-64** | **Age 65-69** |  |  |
| **Age of diabetes diagnosis** | 30-34 | 3 | 3 | 3 | 3 | 3 | 3 | → |  |
|  | 35-39 | 2 | 2 | 2 | 2 | 2 | 2 |  |  |
|  | 40-44 | 1 | 1 | 1 | 1 | 1 | 1 |  |  |
|  | 45-49 | N/A | 0 | 0 | 0 | 0 | 0 |  |  |
|  | 50-54 | N/A | N/A | 0 | 0 | 0 | 0 |  |  |
|  | 55-59 | N/A | N/A | N/A | -1 | -1 | -1 |  |  |
|  | 60-64 | N/A | N/A | N/A | N/A | -2 | -2 |  |  |
|  | 65-69 | N/A | N/A | N/A | N/A | N/A | -3 |  |  |
| **Smoking status** | Non-smoker | -9 | -5 | 0 | 4 | 9 | 13 | → |  |
|  | Current smoker | -2 | 2 | 6 | 9 | 13 | 17 |  |  |
| **Systolic blood pressure (mmHg)** | 100-119 | -1 | -1 | -1 | -1 | -1 | 0 | → |  |
|  | 120-139 | 1 | 1 | 1 | 1 | 1 | 0 |  |  |
|  | 140-159 | 3 | 3 | 3 | 2 | 2 | 1 |  |  |
|  | ≥160 | 6 | 5 | 4 | 4 | 3 | 2 |  |  |
| **Total cholesterol (mmol/L)** | 3.0-3.9 | -4 | -4 | -3 | -3 | -3 | -2 | → |  |
|  | 4.0-4.9 | -3 | -2 | -2 | -2 | -2 | -1 |  |  |
|  | 5.0-5.9 | -1 | -1 | -1 | -1 | -1 | 0 |  |  |
|  | 6.0-6.9 | 1 | 1 | 1 | 1 | 1 | 0 |  |  |
|  | ≥7.0 | 3 | 3 | 2 | 2 | 2 | 1 |  |  |
| **HDL cholesterol (mmol/L)** | 0.5-0.9 | 2 | 1 | 1 | 1 | 1 | 1 | → |  |
|  | 1.0-1.4 | 0 | 0 | 0 | 0 | 0 | 0 |  |  |
|  | ≥1.5 | -1 | - 1 | -1 | -1 | -1 | -1 |  |  |
| **HbA1c (mmol/mol)** | 30-39 | 1 | 1 | 0 | 0 | 0 | 0 | → |  |
|  | 40-49 | 2 | 2 | 2 | 2 | 1 | 1 |  |  |
|  | 50-59 | 4 | 3 | 3 | 3 | 2 | 2 |  |  |
|  | 60-69 | 5 | 5 | 4 | 4 | 3 | 3 |  |  |
|  | ≥70 | 7 | 6 | 5 | 5 | 4 | 4 |  |  |
| **eGFR (mL/min/1.73 m²)** | 30-44 | 8 | 7 | 6 | 6 | 5 | 4 | → |  |
|  | 45-59 | 4 | 4 | 3 | 3 | 3 | 2 |  |  |
|  | 60-89 | 1 | 1 | 1 | 1 | 1 | 1 |  |  |
|  | ≥90 | -1 | -1 | -1 | 0 | 0 | 0 |  |  |
| **Points total** | | | | | | | | |  |

*N/A – Not applicable, mmHg – Millimetres of mercury, mmol/L – Millimoles per litre, HDL – High-density lipoprotein cholesterol, HbA1c – Glycated haemoglobin, mmol/mol – Millimoles per mole, eGFR – Estimated glomerular filtration rate, mL/min/1.73 m² – Millilitres per minute per 1.73 square metres, → – Indicates continuation across age columns, Points total – Calculated by summing points across all predictors for an individual.*

# **Table S2 -** SCORE2-DIABETES for Women (1)

| **Points table for women with diabetes and current age 40-69** | | | | | | | |  | **Reported points by age column** |
| --- | --- | --- | --- | --- | --- | --- | --- | --- | --- |
| **Risk predictor** | **Risk predictor category** | **Age 40-44** | **Age 45-49** | **Age 50-54** | **Age 55-59** | **Age 60-64** | **Age 65-69** |  |  |
| **Age of diabetes diagnosis** | 30-34 | 4 | 4 | 4 | 4 | 4 | 4 | → |  |
|  | 35-39 | 3 | 3 | 3 | 3 | 3 | 3 |  |  |
|  | 40-44 | 2 | 2 | 2 | 2 | 2 | 2 |  |  |
|  | 45-49 | N/A | 1 | 1 | 1 | 1 | 1 |  |  |
|  | 50-54 | N/A | N/A | -1 | -1 | -1 | -1 |  |  |
|  | 55-59 | N/A | N/A | N/A | -2 | -2 | -2 |  |  |
|  | 60-64 | N/A | N/A | N/A | N/A | -3 | -3 |  |  |
|  | 65-69 | N/A | N/A | N/A | N/A | N/A | -4 |  |  |
| **Smoking status** | Non-smoker | -11 | -6 | 0 | 5 | 11 | 16 | → |  |
|  | Current smoker | -1 | 3 | 8 | 12 | 16 | 21 |  |  |
| **Systolic blood pressure (mmHg)** | 100-119 | -1 | -1 | -1 | -1 | -1 | -1 | → |  |
|  | 120-139 | 1 | 1 | 1 | 1 | 1 | 1 |  |  |
|  | 140-159 | 3 | 3 | 3 | 2 | 2 | 2 |  |  |
|  | ≥160 | 5 | 5 | 4 | 4 | 3 | 3 |  |  |
| **Total cholesterol (mmol/L)** | 3.0-3.9 | -5 | -4 | -4 | -3 | -3 | -2 | → |  |
|  | 4.0-4.9 | -3 | -2 | -2 | -2 | -2 | -1 |  |  |
|  | 5.0-5.9 | -1 | -1 | -1 | -1 | -1 | 0 |  |  |
|  | 6.0-6.9 | 1 | 1 | 1 | 1 | 1 | 0 |  |  |
|  | ≥7.0 | 3 | 3 | 3 | 2 | 2 | 1 |  |  |
| **HDL cholesterol (mmol/L)** | 0.5-0.9 | 2 | 2 | 2 | 2 | 2 | 1 | → |  |
|  | 1.0-1.4 | 0 | 0 | 0 | 0 | 0 | 0 |  |  |
|  | ≥1.5 | -2 | -2 | -2 | -1 | -1 | -1 |  |  |
| **HbA1c (mmol/mol)** | 30-39 | 1 | 1 | 1 | 1 | 0 | 0 | → |  |
|  | 40-49 | 3 | 2 | 2 | 2 | 2 | 1 |  |  |
|  | 50-59 | 5 | 4 | 4 | 3 | 3 | 2 |  |  |
|  | 60-69 | 7 | 6 | 5 | 5 | 4 | 3 |  |  |
|  | ≥70 | 9 | 8 | 7 | 6 | 5 | 4 |  |  |
| **eGFR (mL/min/1.73 m²)** | 30-44 | 9 | 8 | 7 | 6 | 5 | 4 | → |  |
|  | 45-59 | 5 | 5 | 4 | 3 | 3 | 2 |  |  |
|  | 60-89 | 2 | 1 | 1 | 1 | 1 | 1 |  |  |
|  | ≥90 | -1 | -1 | -1 | 0 | 0 | 0 |  |  |
| **Points total** | | | | | | | | |  |

*N/A – Not applicable, mmHg – Millimetres of mercury, mmol/L – Millimoles per litre, HDL – High-density lipoprotein cholesterol, HbA1c – Glycated haemoglobin, mmol/mol – Millimoles per mole, eGFR – Estimated glomerular filtration rate, mL/min/1.73 m² – Millilitres per minute per 1.73 square metres, → – Indicates continuation across age columns, Points total – Calculated by summing points across all predictors for an individual.*

**Table S3.** Lipid-lowering medications

| Statins | These are the primary agents for lowering LDL-C levels in diabetic patients whose LDL-C levels are above target. According to the 2024 ADA Standards of Care, the LDL-C goal is <70 mg/dL for adults with diabetes who are at high cardiovascular risk, and <55 mg/dL for those with established atherosclerotic cardiovascular disease (secondary prevention). |
| --- | --- |
| Statins + Ezetimibe | This combination is recommended if LDL-C targets are not met with statin therapy alone. |
| Statins + Icosapent | This combination is recommended for patients with hypertriglyceridemia. |
| PSCK9 inhibitor | Recommended for patients at very high cardiovascular risk who have persistently elevated LDL-C levels despite being on the maximum tolerated dose of statins combined with ezetimibe.  PSCK9 inhibitor alone recommended for people who are intolerant to statins. |
| PSCK9 inhibitor + Ezetimibe | This combination is advised if statins are not tolerated at any dose, even after re-challenge, and LDL-C targets are not met on PCKS9 inhibitor alone.. |
| Ezetimibe | Recommended as monotherapy in patients who are intolerant to statins or have contraindications to statin use. It may also be considered in selected cases where statin therapy is not feasible. |

# *LDL-C, Low-Density Lipoprotein Cholesterol; PCSK9, Proprotein Convertase Subtilisin/Kexin Type 9; TG, Triglycerides.*

# **Table S4.** Key clinical and laboratory evaluations for chronic kidney disease complications in T2DM

| **Complication** | **Physical and laboratory evaluation** |
| --- | --- |
| **High Blood Pressure (>130/80 mmHg)** | Monitor blood pressure and assess weight |
| **Volume Overload** | Review patient history, conduct physical examination, and check weight |
| **Electrolyte Imbalances** | Test serum electrolyte levels |
| **Metabolic Acidosis** | Test serum electrolyte levels |
| **Anemia** | Measure hemoglobin; conduct iron studies if needed |
| **Metabolic bone disease** | Assess serum levels of calcium, phosphate, parathyroid hormone (PTH), and vitamin 25(OH)D |
| *Complications arising from chronic kidney disease (CKD) generally become apparent when the estimated glomerular filtration rate (eGFR) decreases below 60 mL/min/1.73 m², corresponding to stage 3 CKD or higher. These complications tend to become more frequent and severe as CKD advances. Assessment of elevated blood pressure and volume overload should be performed at every possible clinical encounter. Laboratory evaluations are typically recommended every 6-12 months for stage 3 CKD, every 3-5 months for stage 4 CKD, and every 1-3 months for stage 5 CKD. More frequent testing may be necessary to evaluate symptoms or in response to changes in therapy. mmHg – Millimetres of mercury, PTH – Parathyroid hormone, 25(OH)D – 25-hydroxyvitamin D, CKD – Chronic kidney disease, eGFR – Estimated glomerular filtration rate, mL/min/1.73 m² – Millilitres per minute per 1.73 square metres* | |

*.*

**Figure S1:** Type 2 diabetes pathophysiology

*GLP-1, Glucagon-Like Peptide-1; GIP, Glucose-Dependent Insulinotropic Polypeptide;* *β-Cell – Beta cell (pancreatic cell that secretes insulin); α-Cell – Alpha cell (pancreatic cell that secretes glucagon).*

# **Figure S2.** Illustration of targeted daily dose calculation of basal-bolus insulin regimen.

*TDD, Total Daily Dose; U, Units.*

**Figure S3.** Simplified Guide to Glycemic Targets in Type 2 Diabetes with Cardiovascular Disease.

*^a^Recommended medications include SGLT2 inhibitors (empagliflozin, canagliflozin, dapagliflozin, sotagliflozin) or GLP-1 RAs (liraglutide, semaglutide s.c., dulaglutide, efpeglenatide).^b^Adjust glycemic targets if hyperglycemic symptoms (such as polyuria and polydipsia) are present. ^c^Hypoglycemia concerns primarily apply to patients using sulfonylureas and/or insulin. CV, Cardiovascular; HbA1c, Hemoglobin A1c.*

**Figure S4.** Screening and diagnosis of hypertension in patients with diabetes.

*BP, Blood Pressure; SBP, Systolic Blood Pressure; DBP, Diastolic Blood Pressure; ABPM, Ambulatory Blood Pressure Monitoring; HBPM, Home Blood Pressure Monitoring; mmHg, Millimeters of Mercury.*

**Figure S5.** Glycemic management for people with diabetes and DKD.

*CKD, Chronic Kidney Disease; eGFR, Estimated Glomerular Filtration Rate; ACR, Albumin-to-Creatinine Ratio; ACEi, Angiotensin-Converting Enzyme Inhibitor; ARB, Angiotensin Receptor Blocker; SGLT2i, Sodium–Glucose Cotransporter-2 Inhibitor; GLP-1 RA, Glucagon-Like Peptide-1 Receptor Agonist; CVD, Cardiovascular Disease; A1C, Glycated Hemoglobin; DPP-4, Dipeptidyl Peptidase-4; TZD, Thiazolidinedione; T2D, Type 2 Diabetes.*

**Figure S6.** Algorithm for prevention of diabetic kidney disease progression.

*ACEi, Angiotensin-Converting Enzyme Inhibitor; ARB, Angiotensin Receptor Blocker; eGFR, Estimated Glomerular Filtration Rate; SGLT2i, Sodium–Glucose Cotransporter-2 Inhibitor; GLP-1 RA, Glucagon-Like Peptide-1 Receptor Agonist; L, Liter; m², Square Meter; mmol/L, Millimoles per Liter; mg/day, Milligrams per Day.*

**References**

1.	SCORE2-Diabetes: 10-year cardiovascular risk estimation in type 2 diabetes in Europe. Eur Heart J. 2023;44(28):2544-56.
